# Portraying Persons Who Inject Drugs Recently Infected with Hepatitis C Accessing Antiviral Treatment: A Cluster Analysis

**DOI:** 10.1155/2014/631481

**Published:** 2014-10-01

**Authors:** Jean-Marie Bamvita, Elise Roy, Geng Zang, Didier Jutras-Aswad, Andreea Adelina Artenie, Annie Levesque, Julie Bruneau

**Affiliations:** ^1^CRCHUM (Centre de Recherche du Centre Hospitalier de l'Université de Montréal), Tour Saint-Antoine 850, Rue St-Denis, Montréal, QC, Canada H2X 0A9; ^2^Département de Médecine Familiale, Faculté de Médecine, Université de Montréal, Pavillon Roger-Gaudry, Bureau S-711, 2900 boul. Édouard-Montpetit, Montréal, QC, Canada H3T 1J4; ^3^Faculté de Médecine et des Sciences de la Santé, Université de Sherbrooke, Campus Longueuil 1111, Rue St-Charles Ouest, Bureau 500, Longueuil, QC, Canada J4K 5G4; ^4^Family Medicine Department, McGill University, 5858 Chemin de la Côte des Neiges, 3e Étage, Montréal, QC, Canada H3S 1Z1

## Abstract

*Objectives*. To empirically determine a categorization of people who inject drug (PWIDs) recently infected with hepatitis C virus (HCV), in order to identify profiles most likely associated with early HCV treatment uptake. *Methods*. The study population was composed of HIV-negative PWIDs with a documented recent HCV infection. Eligibility criteria included being 18 years old or over, and having injected drugs in the previous 6 months preceding the estimated date of HCV exposure. Participant classification was carried out using a TwoStep cluster analysis. *Results*. From September 2007 to December 2011, 76 participants were included in the study. 60 participants were eligible for HCV treatment. Twenty-one participants initiated HCV treatment. The cluster analysis yielded 4 classes: class 1: *Lukewarm health seekers dismissing HCV treatment offer*; class 2: *multisubstance users willing to shake off the hell*; class 3: *PWIDs unlinked to health service use*; class 4: *health seeker PWIDs willing to reverse the fate*. *Conclusion*. Profiles generated by our analysis suggest that prior health care utilization, a key element for treatment uptake, differs between older and younger PWIDs. Such profiles could inform the development of targeted strategies to improve health outcomes and reduce HCV infection among PWIDs.

## 1. Introduction

The prevalence of HCV infection is estimated at 130–170 million people worldwide, currently driven by the growing number of infections among people who inject drugs (PWID) [1]. If not treated, the majority (75–85%) evolve to chronic infection; and some (20%) develop intractable and lethal diseases (cirrhosis, liver failure, and hepatoma) [2].

Before the advent of well-tolerated, orally administered HCV treatment regimens, traditional interferon-based antiviral treatment induced significant side effects that were deterring some patients from completing the treatment course. For patients who achieved sustained viral response equivalent to a cure, HCV treatment was shown to bring additional benefits, such as reduction of risky drug-consumption behaviours [3] and improvement of quality of life [4]. It is likely that, within the next three to five years, well-tolerated, orally administered interferon-free regimens will be available, thus improving the feasibility of treating difficult populations [5]. A recent modeling study by Martin and colleagues suggested that significant decreases in HCV prevalence can be accomplished by increasing simultaneously needle exchange program and opiate substitution therapy coverage on the one hand and HCV treatment coverage on the other hand [6]. In large observational community-based drug users' cohorts, however, the HCV treatment uptake was estimated at <8% or less than 1% annually [7]. Further, despite increasing efforts to attract vulnerable population in treatment, the number of PWIDs treated annually still stagnates [8].

Barriers to HCV treatment were found to be multifactorial and included factors impeding optimal access at the level of the patient, system, and practitioner [7]. Attempts to frame the influence of multidimensional factors and conditions facilitating or impeding health care access and outcomes can be guided by the Behavioral Model of Health Services Utilization, a conceptual framework developed by Andersen [9]. Reasons cited by PWIDs with HCV for not seeking treatment include poor education about their condition and its treatment, an absence of noticeable symptoms, fear of adverse effects of treatment, and other ongoing medical comorbidities and social issues [10]. Beyond individual barriers, factors affecting treatment uptake include financial coverage, housing stability, and assessment by the physician of the risks and benefits of immediate versus delayed treatment for HCV-chronically infected individuals [7]. From a service development perspective, it is important to identify profiles of individuals according to treatment uptake. Such profiles could help inform novel interventions to increase treatment uptake in subgroups with specific characteristics. PWIDs recently infected by HCV who are systematically offered treatment under universal financial coverage represent a unique group to study in order to assess how individual profiles, as opposed to specific risk factors, affect treatment uptake. Cluster analysis has been used in intervention research to unmask unknown heterogeneity between concurrent groups by focusing more on inherent differences between cases than on individual variables [11].

The objective of this study was to empirically identify profiles associated with early HCV treatment uptake among recently HCV infected PWIDs who were systematically offered HCV treatment and were covered by universal health insurance.

## 2. Methods

### 2.1. Study Population

The study population was composed of PWIDs recently infected with HCV, enrolled in IMPACT, a study aiming at examining the effect of acute HCV infection and antiviral treatment on the behaviors and quality of life of PWIDs who have access to specific targeted health services. Eligibility criteria included being 18 years old or over, having injected drugs in the previous 6 months or in the 3-month-period preceding the estimated date of HCV infection, and living in the Greater Montreal area. Documented acute HCV infection was defined as either (1) a HCV antibody negative test, followed by either a HCV antibody or RNA positive test within 6 months of the HCV antibody negative test period or (2) an acute symptomatic infection with evidence of hepatitis illness (i.e., jaundice or alanine aminotransferase (ALT) elevation over 400 U/L). Participants were recruited from two main sources: (i) the St. Luc Cohort, a prospective cohort study with semiannual visits designed to examine individual and contextual factors associated with HCV and HIV infections among current PWIDs (i.e., drug injection in the six months prior to recruitment) [12] and (ii) community and hospital-based collaborating clinics, including the addiction medicine clinic at the Centre Hospitalier de l'Université de Montréal (CHUM).

Eligible individuals were invited to participate in the study and were systematically referred to the CHUM addiction medicine clinic for clinical assessment. PWIDs recently infected with HCV, who did not resolve spontaneously after 20 weeks of estimated infection, were offered HCV treatment regardless of their drug use or social conditions.

The research protocol has been approved by the Institutional Research Ethical Board of the CHUM and includes an authorization to access participants' clinical data, when available. A $30 stipend for travel costs was offered for each completed research visit.

### 2.2. Variables and Measurement Instruments

The variable of interest was “treatment initiation,” defined as receiving a first dose of pegylated interferon. Information was retrieved from the clinical chart and validated with the clinical nurse. Two measurement instruments were used to characterize participants. The SF-36 questionnaire was used to assess health related quality of life (QualityMetric Health Outcomes Scoring Software 4.0). This questionnaire has been extensively used and validated in various patient settings and in the general population [13]. Using factor analysis, items of this questionnaire are conceptually reduced to two main dimensions: physical and mental component of quality of life, which were used for analysis in this study. A short interviewer-administered questionnaire, derived from the St. Luc Cohort questionnaire [14], was used to collect sociodemographic characteristics, information on injection drug use practices, health related factors, and service utilization. Drug use consumption was documented for the prior 6 months.

Given the focus on healthcare utilization, the sample has been described according to the Andersen model, with variables categorized as predisposing, enabling, and need factors [9]. Predisposing factors comprise individual variables associated with service utilization. Enabling factors include contextual, systemic, or structural variables associated with service utilization. Need factors relate to diseases or risky behaviors that could impact on health and well-being. Variables considered in our model were further chosen with respect to the current body of knowledge on HCV treatment access for drug users.

### 2.3. Analyses

Frequency distribution for categorical variables and mean values along with standard deviations for continuous variables were used for descriptive analyses. Bivariate analyses using Pearson chisquare statistics for categorical variables and independent sample *t*-test for continuous variables were conducted to compare PWID characteristics according to HCV treatment initiation. Statistically significant differences were assessed at *P* < 0.05; *P* values were two-sided.

Participant profile was carried out by means of a TwoStep cluster analysis using SPSS Statistics 20.0 package [15, 16]. Variables were introduced in the cluster analysis in an orderly manner, categorical variables first and then continuous variables. The first categorical variable entered was “having initiated HCV treatment.” Age categories and housing categories were multicategorical variables. The SF-36 physical and mental component scores were entered as continuous scores in the model. The log-likelihood method was used to determine intersubject distance. The first iteration yielded a two-class cluster model based on Schwarz Bayesian criteria and log-likelihood method, reflecting the overall contribution of participants to the interclass homogeneity. This cluster analysis was discarded because classes were not contrasted enough for interpretation [17]. Finally the number of classes was set at 4 and produced an acceptable model. The quality of the model was estimated as satisfactory by the class cohesion and separation test.

## 3. Results

From September 2007 to December 2011, 76 participants infected with HCV within the previous six months were recruited in Montreal, Canada. Sixteen (21%) cleared their infection spontaneously and were not included in this investigation.  Table 1 presents descriptive characteristics of the 60 participants included in analyses, along with comparison analyses between those who have initiated HCV treatment and those who have not. Overall, 21 participants (35%) had initiated HCV treatment.

The four-class cluster analysis is displayed on Table 2. Classes were labelled according to the most prominent characteristics within classes. The four classes can be described as follows.

*Class 1. Lukewarm Health Seekers Dismissing HCV Treatment Offer*. This includes younger participants (79% under 30 years old), mostly females (86%), poorly educated (93% without a college degree), living predominantly in stable housing (64%). Compared to other classes, they rank fourth as to cocaine injection (64%) and second as to heroin injection. They have the lowest score on both physical and mental components of quality of life. They represent one of the two highest proportions of participants followed up by a family physician (35%) and the third lowest proportion of HCV treatment uptake (14%).

*Class 2. Multisubstance Users Willing to Shake off the Hell*. This includes mostly younger participants (87% under 30 years old), exclusively males, poorly educated, living mostly in stable housing. All members (100%) of this class use IV cocaine and IV heroin. They rank first as regard alcohol consumption and have the highest proportion of methadone program involvement. 53% have initiated a HCV treatment, ranking second of the 4 classes. 

*Class 3. PWIDs Unlinked to Health Service Use*. This includes middle-age participants (64% between 30 and 40 years old), exclusively males, with the highest proportion of homelessness of all classes, injecting mostly cocaine. They also report the lowest involvement in health service use. No one in that class has initiated a HCV treatment. 

*Class 4. Health Seeker PWIDs Willing to Reverse the Fate*. This includes the oldest group (all over 30 years old), mostly males, poorly educated, living predominantly (90%) in unstable housing conditions and using IV cocaine use. Participants in this class have the highest score on the physical component of quality of life, the highest proportion of health service use, and the highest proportion of HCV treatment initiation.

## 4. Discussion

PWIDs face many challenges and experience competing needs when it comes to taking care of their health. Overall, 35% of eligible PWIDs initiated treatment. The proportion of participants treated in our study soon after diagnosis is greater than in most studies among HCV infected active PWIDs [18]. This may indicate that delaying treatment, either for recently or chronically infected individuals, might not be the best option to increase uptake. Findings from a recent clinical trial conducted in Canada support this assumption: a higher overall sustained viral response (65% versus 39%) was found among PWIDs allocated to immediate versus delayed treatment onset [19].

This study was undertaken to draw profiles associated with HCV treatment uptake after recent infection, in a setting where treatment was systematically offered under universal health insurance coverage. Overall, results suggest that educated male and female PWIDs and those who had links with various health care services, as shown by prior hepatitis B vaccination, opiate substitution treatment (OST) participation, and visit to a health care professional, were more likely to initiate HCV treatment after recent infection, regardless of drug-consumption. As in McGowan study [20], participants in classes 2 and 4, who initiated treatment, were also characterized by lower self-rated mental health quality of life. According to Anderson's model, prior healthcare service utilization may enable further health service use [9]. Participants in classes 2 and 4, which together comprise 90% of all participants treated, had higher proportions of methadone program participation, hepatitis B vaccination, and follow-up by family physician. In a study conducted in Australia by Digiusto and Treloar [21], participants who had consulted a general practitioner for medication were more likely to have initiated HCV treatment. Participation to a methadone maintenance treatment has been associated with a higher willingness to be treated [22], to increased treatment uptake [23] and to better outcomes [24]. In a recent study among drug users followed in methadone and community clinics with enhanced HCV treatment access, methadone was not associated with uptake [25].

A salient characteristic of this cluster analysis was the identification of distinct profiles according to treatment uptake, for which standard comparisons were not quite informative. For instance, age was not statistically associated with treatment uptake in bivariate analysis. However, the age distribution in clusters suggests that uptake profiles differ between older and younger drug users. Classes 1 and 2 comprised 24 of the 28 individuals under 30. In contrast, classes 3 and 4 included all but five individuals over 30.

Hence, when contrasting “younger” (classes 1 and 2) and “older” (classes 3 and 4) PWID profiles, results from the cluster analysis suggest that the effect of health care utilization, an important element for treatment uptake, differed between older and younger groups. Younger individuals who initiated treatment reported being in methadone substitution treatment in higher proportions. Vaccination and family physician attendance were reported by a substantial proportion of older individuals initiating treatment, and by none of those who did not. In addition, class profiles showed that housing status, namely, living in a prison, a shelter, or in a therapy setting, was related to treatment uptake among older PWIDs, but not so among younger drug users.

The seemingly positive impact of living in an institutional facility, either prison, therapy, or shelter, on treatment uptake among older participants in our study may indicate enhanced linkages with healthcare services through service providers, relative to other individuals in this cohort [26]. Conversely, class 3 profile includes a majority of homeless individuals, no one having initiated HCV treatment. According to Andersen's theory, when healthcare access is determined by enabling factors, such as their housing situation among older participants, systemic inequity is an issue [9].

Active use of illicit drugs is a treatment barrier documented in many studies. Active illicit drug use was associated with reluctance to initiate HCV treatment by the patient [27] and by the physician [28]. Alcohol abuse was also found associated with not initiating treatment [29]. In our setting, however, the proportion of participants reporting drug and alcohol use was slightly higher among initiates relative to participants who were not treated, consistent across all classes. Active substance use was not a motive to deny treatment in this study. This finding suggests that active drug use may not be an important factor in the decision to get treated in the absence of systemic and practitioner-level barriers. It is also possible that ongoing drug use was linked to more contact with health services, probably due to multiple health related consequences of drug use overtime.

Results of this study are subject to numerous limitations. First, we acknowledge that our sample may not be representative of drug users in other settings. If there have been some observed shifts in its use, cocaine is still the most prevalent injection drug used in Eastern Canada [30]. Moreover, cocaine use worldwide has remained stable, with indications of increases in Oceania, Asia, Africa, and some countries in South America [31]. Despite close clinical follow-up of participants through laboratory analyses, our results could be biased by the self-reported behavioral data related to alcohol and drug use. In general, self-reported data from PWIDs tend to be accurate [32]. This study could also be subject to interviewer bias, which has been mitigated, if not prevented, by regular retraining of interviewers to uphold the integrity of data collection procedures and avoid imposition of systematic bias. A sample of 60 participants is obviously low. Nonetheless, the quality of the model was estimated to be satisfactory.

## 5. Conclusion

This study underscores the importance of reaching beyond the individual-level factors in characterizing vulnerable populations in relation to HCV treatment uptake. Looking at profiles instead of individual variables can help tackle health related behaviors of PWIDs recently infected with HCV. This natural experiment represents a novel approach to understanding how specific patient characteristics can be used to develop targeted strategies to improve health outcomes and reduce HCV infection. For example, systemic barriers should be recognized early among those eligible for HCV treatment—such as difficulty in accessing decent accommodation or job—and tackled strategically by linking patients with case manager and social worker services.

## Figures and Tables

**Table 1:** Characteristics of participants and comparative analyses according to treatment initiation (*n* = 60).

	Frequency distribution						Comparison tests
	Total sample (*N* = 60)		Treatment not initiated *n* = 39 (65%)		Treatment initiated *n* = 21 (35%)		*P* value*
	*n*	%	*n*	%	*n*	%	
Age categories							
<30 years old	28	46.7	21	53.8	7	33.3	
30–39 years old	15	25.0	9	23.1	6	28.6	0.311
>40 years old	17	28.3	9	23.1	8	38.1	0.133
Gender							
Female	15	25.0	11	28.2	4	19.0	0.437
Male	45	75.0	28	71.8	17	81.0	
Education							
Secondary or less	44	73.3	30	76.9	14	66.7	0.397
College or above	16	26.7	9	23.1	7	33.3	
Housing							
Stable housing (home, apartment, room)	25	41.7	18	46.2	7	33.3	
Temporary housing (therapy, prison, shelter)	22	36.7	12	30.8	10	47.6	0.217
Homeless	13	21.7	9	23.1	4	19.0	0.858
Alcohol consumption	36	60.0	23	59.0	13	61.9	0.825
IV drugs consumed							
IV heroine	29	48.3	19	48.7	10	47.6	0.935
IV cocaine	53	88.3	34	87.2	19	90.5	0.705
Vaccines received							
Hepatitis B vaccine	17	28.3	7	17.9	10	47.6	0.015
Quality of life scores							
PCS mean (SD)	46,4	10.2	45.6	9.8	47.9	10.9	0.389
MCS mean (SD)	33,9	13.9	34.0	14.2	33.9	13.8	0.985
Methadone	20	33.3	10	25.6	10	47.6	0.085
Having been followed up in the 6 prior months by a family physician	11	18.3	6	15.4	5	23.8	0.424

*Pearson chi-square.

**Table 2:** Participants typology (cluster analysis; *N* = 60).

	Class 1 *n* = 14 (23.3%)	Class 2 *n* = 15 (25.0%)	Class 3 *n* = 11 (18.3%)	Class 4 *n* = 20 (33.3%)	Combined *N* = 60 (100.0%)
Predisposing factors					
Age categories *n* (%)					
<30 years old	11 (78.6)	13 (86.7)	4 (36.4)	0 (0.0)	28 (46.7)
30–39 years old	3 (21.4)	2 (13.3)	7 (63.6)	3 (15.0)	15 (25.0)
40 years old and over	0 (0.0)	0 (0.0)	0 (0.0)	17 (85.0)	17 (28.3)
Gender *n* (%)					
Females	12 (85.7)	0 (0.0)	0 (0.0)	3 (15.0)	15 (25.0)
Males	2 (14.3)	15 (100.0)	11 (100.0)	17 (85.0)	45 (75.0)
Education *n* (%)					
Elementary/secondary	13 (92.9)	12 (80.0)	6 (54.5)	13 (65.0)	44 (73.3)
College or over	1 (7.1)	3 (20.0)	5 (45.5)	7 (35.0)	16 (26.7)
Enabling factor					
Housing *n* (%)					
Stable housing (home, apartment, room)	9 (64.3)	9 (60.0)	5 (45.5)	2 (10.0)	25 (41.7)
Temporary housing (therapy, prison, shelter)	4 (28.6)	2 (13.3)	0 (0.0)	16 (80.0)	22 (36.7)
Homeless	1 (7.1)	4 (26.7)	6 (54.5)	2 (10.0)	13 (21.7)
Need factors					
IV cocaine consumption *n* (%)	9 (64.3)	15 (100.0)	11 (100.0)	18 (90.0)	53 (88.3)
IV heroine consumption *n* (%)	9 (64.3)	15 (100.0)	2 (18.2)	3 (15.0)	29 (48.3)
Alcohol consumption *n* (%)	8 (57.1)	13 (86.7)	4 (36.4)	11 (55.0)	36 (60.0)
Quality of life (SF-36) (mean (SD)					
PCS mean (SD)	45.7 (6.9)	46.4 (9.4)	46.7 (9.1)	46.8 (13.4)	46.4 (10.2)
MCS mean (SD)	25.3 (12.1)	37.0 (14.8)	37.5 (8.1)	35.7 (15.3)	33.9 (13.9)
Health service utilization					
Methadone program *n* (%)	5 (35.7)	8 (53.3)	3 (27.3)	4 (20.0)	20 (33.3)
Hepatitis B vaccine *n* (%)	4 (28.6)	3 (20.0)	0 (0.0)	10 (50.0)	17 (28.3)
Followed up by a family physician *n* (%)	5 (35.7)	1 (6.7)	0 (0.0)	5 (25.0)	11 (18.3)
Having initiated treatment *n* (%)	**2 (14.3)**	**8 (53.3)**	**0 (0.0)**	**11 (55.0)**	**21 (35.0)**
